# Reliability of flow-cytometry in diagnosis and prognostic stratification of myelodysplastic syndromes: correlations with morphology and mutational profile

**DOI:** 10.1007/s00277-023-05384-2

**Published:** 2023-08-03

**Authors:** Luca Guarnera, Emiliano Fabiani, Cristina Attrotto, Hajro Hajrullaj, Antonio Cristiano, Roberto Latagliata, Susanna Fenu, Francesco Buccisano, Maria Irno-Consalvo, Consuelo Conti, Maria Teresa Voso, Luca Maurillo

**Affiliations:** 1 Department of Biomedicine and Prevention, Tor Vergata University, Rome, Italy; 2 UniCamillus-Saint Camillus International University of Health Sciences, Rome, Italy; 3 Haematology Division, Ospedale Belcolle, Viterbo, Italy; 4 Haematology Department, San Giovanni-Addolorata Hospital, Rome, Italy; 5 Neuro-Oncohematology Unit, Istituto di Ricovero e Cura a Carattere Scientifico (IRCCS) Fondazione Santa Lucia, Rome, Italy

**Keywords:** Myelodysplastic syndromes, Acute myeloid leukemia, Clonal disorders

## Abstract

Diagnosis and prognostic stratification of myelodysplastic syndromes (MDS) have been complemented by new techniques, including flow cytometry and NGS. To analyze the relationship between molecular and cytofluorimetric data, we enrolled in this retrospective study, 145 patients, including 106 diagnosed with MDS and 39 controls. At disease onset, immunophenotypic (IF), cytogenetic tests, and cytomorphological (CM) examination on bone marrow were carried out in all patients, while NGS was performed in 58 cases. Ogata score presented a specificity of 100% and a sensitivity of 59%. The detection of at least two phenotypic aberrancies in Ogata negative patients increased the sensitivity to 83% and specificity to 87%. Correlations were identified between IF aberrancies and mutations, including positive Ogata>2 and mutations in SRSF2 (*p*=0.035), CD15 and U2AF1 (0.032), CD56 and DNMT3A (*p*=0.042), and CD38 and TP53 (*p*=0.026). In multivariate analysis, U2AF1 mutations, associated with del(20q) and/or abnormalities of chromosome 7 (group 4 as defined by the EuroMDS score), significantly correlated with an inferior overall survival (*p*=0.019). These parameters and Ogata score>2 also showed a significant correlation with inferior event-free survival (*p*=0.023 and *p*=0.041, respectively). Both CM and FC features correlated with prognosis and mutational patterns. In an integrated MDS work-up, these tools may guide indications for mutational screening for optimal risk stratification.

**Supplementary Information:**

The online version contains supplementary material available at 10.1007/s00277-023-05384-2.

## Introduction

Myelodysplastic syndromes (MDS) are a heterogeneous group of clonal disorders characterized by persistent peripheral cytopenia, dysplasia of myeloid progenitors, and increased risk of acute myeloid leukemia (AML) [1]. The backbone for establishing diagnosis and prognosis of MDS are a careful anamnesis (to exclude potential other causes of cytopenia), bone marrow (BM), cytomorphological (CM) examination, and cytogenetic tests [2].

Over the years, these valuable tools have been complemented by new techniques for a better characterization and prognosis assessment of MDS. In this line in 2014, the European Leukemia Net (ELN) working group for flow cytometry strongly recommended the use of this technique in the work-up of MDS [3].

Despite the lack of a standardized profile for diagnosis, flow cytometry (FC) has been shown to be reliable in enumerating bone marrow progenitors and CD34+ cells and identifying phenotypic aberrancies, which correlate with transfusion dependence, poor response to therapy, unfavorable cytogenetics, evolution into AML, and decreased overall survival (OS) [3–6]. In clinical practice, a FC score has been proposed by Ogata et al. in 2009 [7] and validated by Della Porta et al. in 2012 [8] for IPSS low-risk MDS, whose diagnosis may be challenging in absence of blasts, ring sideroblasts, or clonal cytogenetic abnormalities.

More recently, the advances in genome sequencing have led to a better understanding of the genomic landscape of MDS, detecting mutations in 78–89% of cases [9–12]. These acquisitions have allowed to create new classifications and predict more accurately response to treatment and prognosis.

In particular, the recently published International Molecular Prognosis Scoring System (IPSS-M) [13], combining genomic profiling with hematologic and cytogenetic parameters, improved risk stratification of patients with primary and secondary or therapy-related MDS. Indeed, by stratifying MDS into 6 risk classes, IPSS-M predicts survival with significantly higher accuracy, as compared to IPSS [14] and revised IPSS (R-IPSS) [15, 16].

Despite these advances, the relationship between molecular and FC data and the diagnostic potential of the combined analysis need to be defined.

In this retrospective study, we propose a FC panel to detect phenotypic aberrancies on CD34+ myeloid progenitors, in combination with the “Ogata score,” to support the diagnosis and prognosis of MDS and explore the correlations with morphologic examination and mutational profile.

## Patients and methods

### Patient characteristics

This retrospective study included 145 patients consecutively diagnosed with MDS from November 2017 to February 2022 at Policlinico Universitario di Roma Tor Vergata and Policlinico Universitario di Roma Umberto I (106 with MDS and 39 with non-clonal cytopenias, as controls). Diagnosis of MDS was made according to 2016 WHO criteria [1]. Patient characteristics are summarized in Table 1.

**Table 1:** Study population

Age, years (*N*=106)	Median (range)	73 (37–89)
Sex (*N*=106)	Male (%)	63 (59)
	Female (%)	43 (41)
WHO 2016 (*N*=106)	MDS with single lineage dysplasia	19 (18)
	MDS with multilineage dysplasia	46 (43)
	MDS with isolated del(5q)	8 (8)
	MDS with ring sideroblasts and single lineage dysplasia	10 (9)
	MDS with excess blasts-1	15 (14)
	MDS with excess blasts-2	7 (7)
	MDS, unclassifiable	1 (1)
R-IPSS* (*n*=105)	Very low (%)	24 (23)
	Low (%)	51 (49)
	Intermediate (%)	11 (10)
	High (%)	17 (16)
	Very high (%)	2 (2)
IPSS-M* (*n*=49)	Very low (%)	2 (4)
	Low (%)	32 (65)
	Moderately low (%)	6 (12)
	Moderately high (%)	2 (4)
	High (%)	6 (12)
	Very high (%)	1 (2)

*Blast count was evaluated by morphology and, if not evaluable, by FC

For each case, potential causes of cytopenia were ruled out, including vitamin or iron deficiency, liver or kidney failure, alcohol intake, heavy metal poisoning, chronic inflammatory diseases, thyroid dysfunction, infectious diseases, immunological or rheumatological causes, autoimmune cytopenias, drug-induced cytopenias, and other hematological disorders.

The threshold to define morphological dysplasia was at least 10% cells of one or more myeloid lineage (erythroid, granulocytic, and megakaryocytic). The R-IPSS and IPSS-M were used to categorize MDS from a prognostic perspective [13, 15]. At the onset of disease, immunophenotypic (IF), cytogenetic tests, and morphologic examination on bone marrow (BM), according to current indications [17–19], were carried out on every sample. A subset of 58 patients underwent next-generation sequencing (NGS).

MDS were defined “lower-risk” when R-IPSS was from very low, to intermediate risk, and IPSS-M from very low to intermediate-low. Higher-risk MDS included high and very high risk by R-IPSS, and intermediate-high to very high risk by IPSS-M.

Patients were also categorized basing on the classification proposed by Bersanelli et al. [12] (EuroMDS score): Group 1, MDS with SF3B1 mutations and co-existing mutations in other genes (ASXL1 and RUNX1); Group 2, MDS with TP53 mutations and/or complex karyotype; Group 3, MDS with SRSF2 and concomitant TET2 mutations; Group 4, MDS with U2AF1 mutations associated with deletion of chromosome 20q and/or abnormalities of chromosome 7; Group 5, MDS with SRSF2 mutations with co-existing mutations in other genes (ASXL1, RUNX1, IDH2, and EZH2); Group 6, MDS with isolated SF3B1 mutations (or associated with mutations of TET2 and/or JAK/STAT pathways genes); Group 7, MDS with AML-like mutation patterns (DNMT3A, NPM1, FLT3, IDH1, and RUNX1 genes); and Group 0, MDS without specific genomic profiles.

### Flow cytometry

Ogata score was performed as described by Della Porta et al. [8] by analysing the following parameters: (1) lymphocyte to myeloblast CD45 ratio (mean fluorescence intensity [MFI] of CD45 on lymphocytes ÷ MFI of CD45 on CD34+myeloblasts); (2) granulocyte to lymphocyte SSC peak channel ratio (SSC channel number where the maximum number of CD10– granulocytic cells occurs ÷ SSC channel number where the maximum number of lymphocytes occurs); (3) the percentage of CD34+ B-progenitor-related cells in all CD34+ cells; and (4) the percentage of CD34+ myeloblasts in all nucleated cells. One point was given for each parameter outside the normal values. The analysis of phenotypic aberrancies was performed on gated CD34+ cells evaluating: abnormal expression of CD200 and CD25, lack or reduced expression of CD117, HLA-DR, CD33, CD38, and CD13; asynchronous expression of CD15 and CD64; and cross lineage expression of CD7, CD2, CD5, and CD56 (Supplementary table 1). Supplementary table 2 shows the monoclonal antibody panels used for immunophenotypic testing.

### Molecular screening

Bone marrow mononuclear cells (BM-MNC) were isolated from diagnostic samples of MDS patients by Ficoll gradient centrifugation using lympholyte-H (Cedaralane).

DNA samples were extracted using the QIAamp DNA Mini Kit (Qiagen AG, Milan, Italy), in accordance with the manufacturer’s instructions and quantified using a Qubit Fluorometer (Life Technologies). DNA samples for NGS screening were processed and analyzed as previously reported [20]. In brief, NGS screening for common somatic mutations in thirty genes known to be involved in MDS pathogenesis (supplementary table 3) was performed according to the commercial Myeloid Solution by SOPHiA GENETICS (SOPHiA GENETICS, Saint-Sulpice, Switzerland) on a MiniSeq® sequencing platform (Illumina, San Diego, California). The NGS analysis was performed on generated FASTQ sequencing files using the SOPHiA DDM® platform that allows for detection, annotation, and pre-classification of genomic mutations (SNVs and Indels) through its SOPHiA™ artificial intelligence. Reads were aligned to the human reference genome (hg19 assembly). Only mutations with a VAF ≥ 2% (variant allele frequency), threshold coverage ≥ 1000x, and identified as highly or potentially pathogenic by the SOPHiA DDM® platform were considered for all subsequent steps of the analysis. Single-nucleotide polymorphisms (SNP), variants localized in the intronic and UTR regions, and synonymous variants were also excluded from the analysis. Targeted-NGS sequencing data are stored at https://www.sophiagenetics.com (SOPHiA DDM platform) and can be extracted using the Sophia-DDM-v4 password-protected software. Raw data will be provided to researchers upon request to the corresponding author.

### Statistical analysis

Univariate and multivariate analyses were used to establish the connections between the variables. Categorical variables were compared using the chi-square test; odds ratios (OR) with 95% confidence intervals (CI) were also calculated. For expected cell values less than 5, Fisher’s exact test and the exact limits for confidence intervals were preferred. The independent test or Mann-Whitney test was used for continuous variables as appropriate. A *p* value less than 0.05 was considered significant. Sensitivity [true positives (TP)/TP + false negatives (FN)] and specificity [true negatives (TN)/TN + false positives (FP)] were used to determine the diagnostic power and reliability of the scores.

Survival functions were computed with the Kaplan-Meier method; significance was established with log-rank test. OS was defined as survival from date of first diagnosis, while event-free survival (EFS) indicated time from initial diagnosis and disease progression to HR-MDS, AML, or death by any cause. Cox regression was used to determine significant independent prognostic factors affecting survival. Statistical analysis was performed through IBM SPSS Statistics 27 (IBM Corp. in Armonk, NY).

## Results

### Flow cytometry and morphology

In this paper, we show that Ogata score >2 was able to identify 63 of 106 MDS patients without misclassifying any control, resulting in 59% sensitivity and 100% specificity. The analysis of CD34+ cells revealed a median of two phenotype aberrations per patient (range 0–7, supplementary fig. 1), with lack of CD33 expression being the most commonly detected (44%, Supplementary fig. 2).

In the subset of patients with a negative Ogata score (score 0–1, 43/106 MDS, 40%), at least 2 aberrancies, among those investigated in the FC panel (as listed in supplemental table 1), were detected in 58% of cases (25/43). This increased the sensitivity of the combined analysis to 83% (TP 88/106), and the specificity to 87% (true negative, TN 34/39).

The sensitivity of Ogata score, in the subgroup of lower-risk MDS, was 51% and 47.5% (true positive, TP: 44/86 and 19/40, respectively), while, as expected, sensitivity in higher risk MDS was 100% and 78% (TP 19/19 and 7/9, according to R-IPSS and IPSS-M, respectively.

Moreover, in Ogata score ≤1 lower-risk MDS (R-IPSS: 42/86, 49%; IPSS-M 21/40, 52.5%) the detection of at least 2 phenotypic aberrancies markedly increased the reliability of the tool, bringing the sensitivity to 79% (R-IPSS, 24/42 patients) and 75% (IPSS-M, 11/21 patients), despite lowering specificity to 87% in both groups (TN 34/39).

The mean FC blast count (CD34+ blasts) was 2.2% (median 0.9, range 0–12). We identified a correlation between higher blast counts and specific FC aberrancies (CD38, *p*=0.002, *R*=0.357; CD56, *p*=0.024, *R*=0.101) and Ogata score ≥2 (*p*<0.001, *R*=0.424) (Supplementary fig. 3).

To assess the predictive role of FC in our patient cohort, we performed an outcome analysis, which revealed a correlation between Ogata score ≥2 and higher FC blast count with AML evolution (*p*=0.001 and *p*=0.024, respectively), shorter OS (*p*=0.004 and *p*=0.002, respectively), and event-free survival (*p*=0.003 and *p*=0.001, respectively). Furthermore, we found a correlation with higher R-IPSS (both *p*<0.001), while IPSS-M did not reach the significance threshold.

CM blast count was performed in 92 patients (mean blast count 3.1; median 2, range 0–15). Higher blast counts correlated with specific FC aberrancies (CD15, *p*=0.024, *R*=0.271; CD56, *p*=0.006, *R*=0.156), and a positive Ogata score (*p*=0.007, *R*=0.338) (Supplementary fig. 3), progression to AML (*p*=0.001), shorter EFS (*p*<0.001), OS (*p*<0.001), and higher R-IPSS (*p*<0.001), but not with IPSS-M.

Blast enumerations through FC and CM were strongly correlated (*p*<0.001). We then compared the two techniques subgrouping the patients according to blast counts (group 1: <5% blasts, group 2: ≥5% and <10%, and group 3: ≥10%). Using FC, 78 (85%) patients were assigned to group 1, 9 (10%) to group 2, and 5 (5%) to group 3, while using CM, 71 (77%) patients were assigned to group 1, 15 (16%) to group 2, and 6 (6%) to group 3. The concordance index was 81.5% (Supplementary fig. 4).

### Mutational screening

Using NGS, we detected a total of 81 pathogenic/likely pathogenic mutations in 38 out of 58 patients, (65.5%; median number of mutation per patient: 1, range 0–6) (Supplementary table 4). The median number of mutated genes per patient was 1 (range 0–5).

**Fig. 1:**
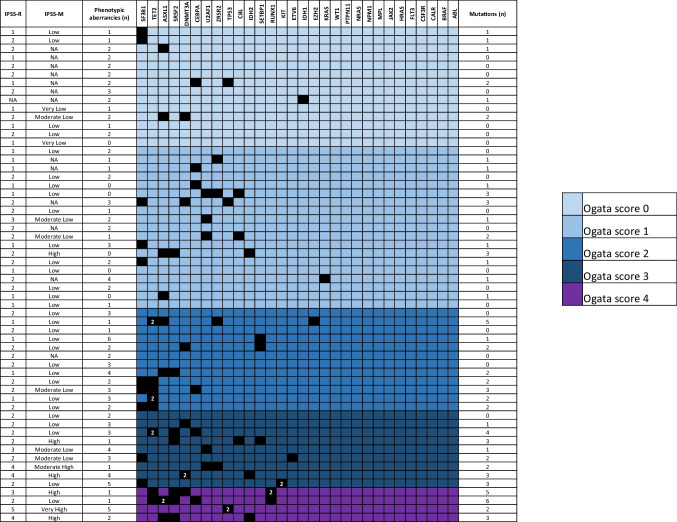
Mutational profiles of 58 patients, grouped according to Ogata score, with information on R-IPSS and IPSS-M

The most frequently mutated genes were SF3B1 (17%, 10/58 patients), ASXL1 (14%, 8/58 patients), and TET2 (14%, 8/58 patients). Figure 1 shows the distribution of mutations.

In lower risk R-IPSS, 33 out of 53 patients (62%) presented at least 1 mutation, while in R-IPSS higher-risk subgroups all 4 patients were mutation carriers (100%; *p*=ns). As previously described [9], the detection of a high number of mutations (≥2) correlated with shorter OS (*p*=0.009) and EFS (*p*=0.056) (suppl. Fig. 5).

We then analyzed the subgroups of patients with mutation in splicing (SF3B1, SRSF2, U2AF1, and ZRSR2; 21/58 patients, 36%) and epigenetics modifying genes (DNMT3A, IDH1, IDH2, and TET2; 16/58 patients, 27.5%). The former presented significantly lower OS (*p*=0.004) and EFS (*p*=0.005).

Supplementary table 4 shows the genomic groups according to the classification proposed by Bersanelli et al. [12]. The most frequently represented was group 0 (MDS without specific genomic profiles; 48%) in agreement with the high prevalence of low-risk-MDS. Group 4 (MDS with U2AF1 mutations associated with deletion of chromosome 20q and/or abnormalities of chromosome 7) presented shorter OS (*p*<0.001) and EFS (*p*<0.001), as previously reported [12].

### Correlation between flow cytometry and mutational screening

The detection of >2 mutations correlated with Ogata score ≥2 (*p*=0.001, OR 6.750; 95% CI: 2.048–22.250) (Supplementary fig. 6), but not with BM blast counts assessed by CM. Number of FC aberrancies did not correlate with number of mutations. However, there were significant associations between FC and genomic data (gene mutations and molecular/prognostic subgroups defined according to Bersanelli et al. [12]).

Ogata score ≥2 was found to be significantly associated with the presence of epigenetic modifier genes as well as SRSF2 and TET2 mutations (*p*=0.003, *p*=0.035, and *p*=0.001, respectively), whereas Ogata score <2 was found to be significantly associated with EuroMDS group 0 (MDS without specific genomic profiles, *p*=0.009). In terms of FC aberrancies, CD56 expression was associated with DNMT3A mutation and EuroMDS group 7 (*p*=0.042 and *p*=0.023, respectively), CD15 expression was associated with U2AF1 mutation and EuroMDS group 4 (*p*=0.032 for both), lower CD117 expression was associated with EuroMDS group 2 (*p*=0.052), and CD38 aberrancy was associated with TP53 mutation (*p*=0.026) (Table 2). A high CM blast count correlated with TP53 mutation (*p*=0.053, *R*=0.439) and group 2 (MDS with TP53 mutations and/or complex karyotype; *p*<0.001, *R*=0.561) whereas a high FC blast count correlated with RUNX1 mutation (*p*=0.024, *R*=0.266) and TP53 mutations (*p*=0.041, *R*=0.315).

**Table 2:** Significant correlations of mutations/EuroMDS score and FC parameters

Correlations between genomic and FC data			
*n* (%)	EuroMDS score	FC	
28 (48%)	Group 0	Ogata score <2	*p*= 0.009, OR: 0.222 95% CI: 0.072–0.684
1 (2%)	Group 2	CD117	*p*= 0.052
5 (9%)	Group 4	CD15	*p*= 0.032, OR: 9.857 95% CI: 1.391–69.835
5 (9%)	Group 7	CD56	*p*= 0.023, OR: 11.750 95% CI: 1.621–85.162
	Mutated gene		
8 (14%)	TET2	Ogata score ≥2	*p*= 0.001
7 (12%)	SRSF2	Ogata score ≥2	*p*= 0.035, OR: 10.105 95% CI: 1.129–90.454
6 (10%)	DNMT3A	CD56	*p*=0.042, OR: 7.667 95% CI: 1.252–46.958
5 (9%)	U2AF1	CD15	*p*=0.032, OR: 9.857 95% CI: 1.391–69.835
3 (5%)	TP53	CD38	*p*= 0.026, OR: 25.500 95% CI: 1.880–345.832

**Fig. 2:**
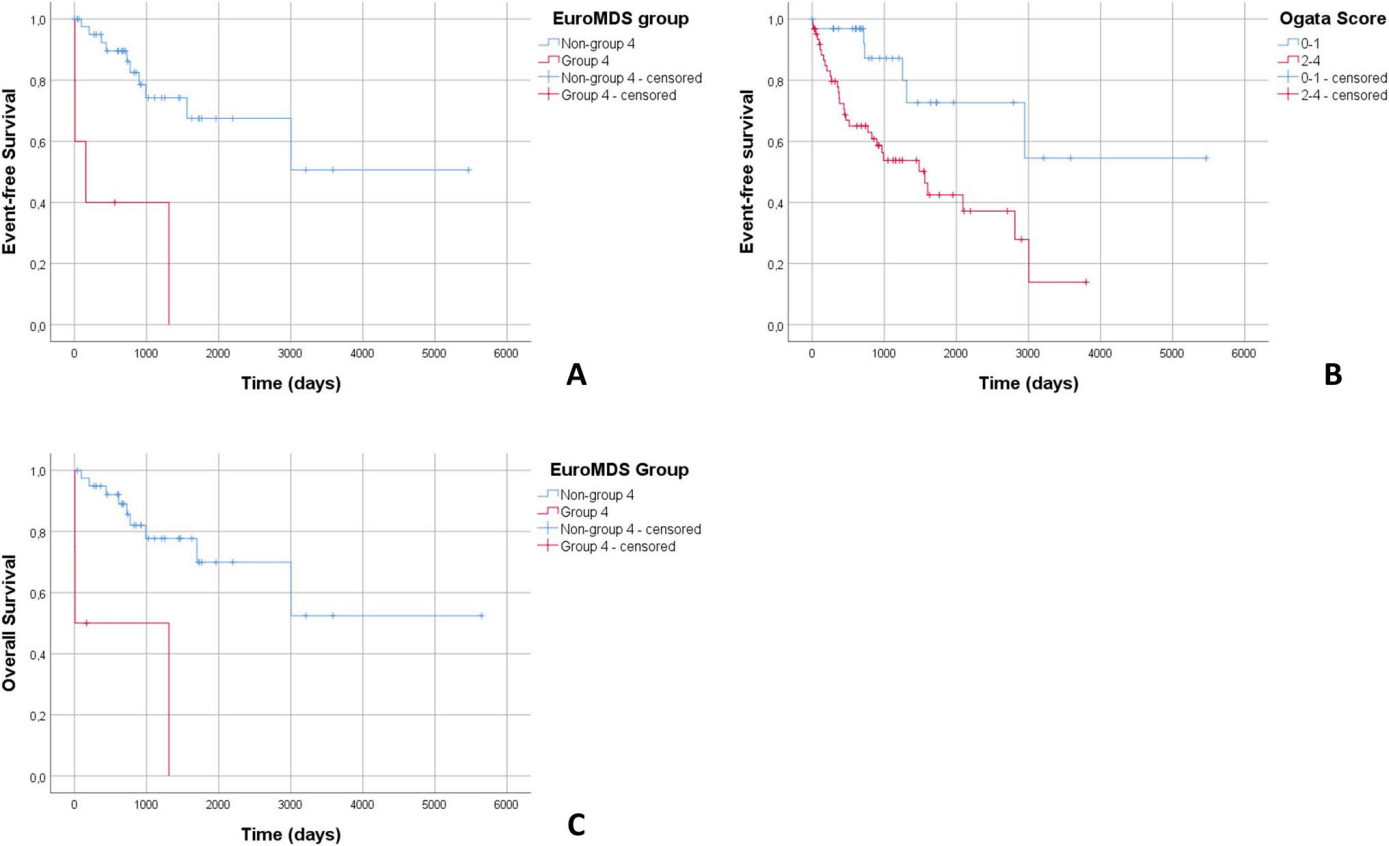
Kaplan-Meier plot of event-free survival (EFS) grouping patients for EuroMDS group 4 (**A**; *p*=0.023 in multivariate analysis) and Ogata score (**B**; *p*=0.041 in multivariate analysis). Kaplan-Meier plot of overall survival (OS) grouping patients for EuroMDS group 4 (**C**; *p*=0.019 in multivariate analysis)

Finally, in multivariate analysis (taking into account age, Ogata score≥2, detection of >2 mutations, CM and FC blast count, mutational subgroup 4 and splicing genes mutations), EuroMDS score group 4 resulted significantly associated with shorter OS (p=0.019). Multivariate analysis of EFS (taking into account age, Ogata score≥2, CM and FC blast count, mutational subgroup 4, and splicing genes mutations) showed a correlation between group 4 and Ogata score ≥2 (*p*=0.023 and *p*=0.041 respectively) (Fig. 2).

## Discussion

The Ogata score provides high specificity when combined with morphology and cytogenetics, as evidenced by our experience (100% specificity). Since its first formulation in 2009, attempts have been made to implement the sensitivity of Ogata score. In Ogata's own paper, a score extended to 7 parameters (CD15, CD56, and CD11b expression on CD34+ myeloblasts) was proposed, which conferred good diagnostic power (sensitivity 72–86%, specificity 90–98%) [7], but it was not validated in a subsequent trial [8].

Other authors proposed the implementation of specific PAs [21, 22] or more complex indices, such as Ki-67 [23], or the study of erythroid precursors [24, 25] or mast-cells [26], with variable results.

Recently, ELN recommended a second-level panel to investigate myeloid progenitor in MDS. Our panel, which differed slightly from the one recommended by ELN [27] due to the inclusion of CD2 and the exclusion of CD11b (see supplementary table 2), confirmed in a real-world setting the value of adding FC parameters to the Ogata score, providing an improvement in sensitivity (83%).

In this regard, specific PAs have been investigated as prognostic factors. Ogata et al., since the early 2000s, identified in several papers CD117, expressed by myeloid progenitors, and CD56 and CD7, usually expressed in other-than myeloid lineages, as markers of higher blast count, evolution to AML and poor prognosis. On the other hand, CD15, whose expression begin to appear in promyelocytes and myelocytes, correlated with a lower blast count and better outcome. In this line, the authors suggested that a more immature immunophenotype could predict an adverse outcome [28–30].

Their interpretation has been questioned in subsequent years, since conflicting evidence about the prognostic role of CD7 emerged: Satoh et al. and Veltroni et al. confirmed the poor prognosis linked to CD7 expression [30, 31], whereas Font et al., analyzing CD34 myeloid cells from 55 bone marrows of patients with MDS, did not detect nor its prevalent expression in higher-risk MDS, neither an association with IPSS or clinical outcome [32, 33].

In our experience, we found a correlation between Ogata Score <2 and Euroscore group 0, that is patients without a specific genetic profile, suggesting that these patients are neither molecularly nor phenotypically well defined, and therefore their characterization remains an unmet medical need. This also questions the diagnosis of MDS in at least some of these patients. In this line, the importance of careful differential diagnosis between MDS, in particular low-risk subtypes, and vitamin deficiencies or cytopenia related to other conditions emerges even more.

The study of potential aberrancies beyond Ogata Score in this setting could be of great help in clarifying the diagnosis and guiding therapeutic. Actually, in our cohort, the detection of 2 phenotypic aberrancies, any of those included in the panel used, resulted highly informative and improved sensitivity in Ogata score-negative patients. In parallel, a broader and more comprehensive phenotypic examination may have a central role in characterize and distinguish pre-malignant conditions, such as idiopathic cytopenia of undetermined significance (ICUS) and clonal cytopenia of undetermined significance (CCUS), in which, again, Ogata Score may not be informative [34]. Further studies are needed to clarify this aspect.

Furthermore, we confirmed the prognostic impact of CD56, as a marker associated with higher blast counts (by both CM and FC analysis) and AML-like mutations patterns (Group 7 and DNMT3A mutations), and the correlation between lower CD117 expression and Bersanelli group 2, characterized by dismal outcome [12], while we did not find any correlations between CD7 and adverse outcome, according to Font et al.

Of note, CD15 expression on CD34+ cells not only did not predict a better outcome in our study cohort, but correlated to higher CM blast counts and to a poor prognosis mutational pattern (U2AF1 mutation and group 4) [12].

Our data confirmed the prognostic value of Ogata Score which, in multivariate analysis, resulted an independent factor significantly associated to EFS.

The comparison between CM examination and FC in blast enumeration is at center of a long-lasting debate. Both techniques are affected by operator dependence and by variables related to blood collection and sample processing. Nevertheless, the strong correlation between the two techniques with a concordance index of 81.5% in blast enumeration found in our study is consistent with previous studies [4]. On the other hand, data in literature about the comparison between FC and CM examination, which, in our experience, tended to identify a higher blast number, are conflicting [4, 35].

Possible explanations could lie in the heterogenicity of the study cohorts and interoperator variability.

The NGS analysis confirmed the negative prognostic role of a high number of mutations, the presence of mutations belonging to splicing-machinery pathway and group 4 identified by Bersanelli et al. [12], whose independent association with OS and EFS is retained, despite taking into account morphological and cytofluorimetric parameters in multivariate analysis. Besides its prognostic value, NGS has been shown to play a role in predicting post hematopoietic cell transplantation outcomes [36] and response to treatments [37, 38].

Thus, in recent years, the use of NGS has increased in clinical laboratories, even outside the field of scientific research [39], although unsolved needs remains, such as the establishment of common quality standards and clinical interpretation of the results. In fact, even if position papers have been published [11, 40], wide variations among laboratories still persist [41]. Furthermore, NGS is still a high-cost technique and low-income countries are struggling to adapt [42]. Moreover, its scarce and uneven availability makes its use complicated even in countries with greater resources.

Therefore, it is necessary to identify the correct subset of patients who could benefit the most from NGS test and the proper panel of genes to analyze to reduce costs and turn around times. According to our findings, specific FC characteristics such as Ogata score and specific PAs correlate with mutational patterns, which can predict outcome and treatment response in the majority of patients. As a result, these low-cost first-line tools could help guide the selection of patients for mutational screening for optimal risk stratification.

### Supplementary information

ESM 1 (DOCX 250 kb)

## Data Availability

The datasets generated during and/or analyzed during the current study are available from the corresponding author on reasonable request.
