# Supplementary material for: Reliability of flow-cytometry in diagnosis and prognostic stratification of myelodysplastic syndromes: correlations with morphology and mutational profile
Source: Ann Hematol. 2023 Aug 3;102(11):3015–23. doi: 10.1007/s00277-023-05384-2 (PMC10567902; doi:10.1007/s00277-023-05384-2)

**Supplementary Tables**

| **Phenotypic aberrancies on CD34+ Myeloid Progenitors** | |
| --- | --- |
| Abnormal expression | CD200*, CD25* |
| Lack or reduced expression | CD117*, HLA-DR*, CD33*, CD13*, CD38* |
| Asinchronous expression | CD15*, CD64* |
| Cross lineage expression | CD7*, CD2*, CD5*, CD56* |

* Each individual phenotypic aberrancy counts as 1 point.

**Supplementary table 1.** Phenotypic aberrancy analysis performed on CD34+ myeloid progenitors.

| **Combination** | **FITC** | **PE** | **PERCP** | **PE-CY7** | **APC** | **APC-H7** | **V450** | **V500** |
| --- | --- | --- | --- | --- | --- | --- | --- | --- |
| **1** | CD10 | CD200 |  | CD19 | CD34 |  |  | CD45 |
| **2** | CD2 | CD56 | CD38 | CD117 | CD34 | CD64 | CD5 | CD45 |
| **3** | CD15 | CD25 | CD7 | CD13 | CD34 | HLA DR | CD33 | CD45 |

APC, allophycocyanin; FITC, fluorescein isothiocyanate; PE, phycoerythrin; PERCP, peridinin clorophyll protein.

**Supplementary table 2.** Panels of monoclonal antibodies used for immunophenotypic tests.

| **Gene** | **Target region (exon)** | **Gene** | **Target region (exon)** | **Gene** | **Target region (exon)** |
| --- | --- | --- | --- | --- | --- |
| *ABL* | 4-9 | *FLT3* | 13-15 and 20 | *PTPN11* | 3,7-13 |
| *ASXL1* | 9,11,12 | *HRAS* | 2,3 | *RUNX1* | all |
| *BRAF* | 15 | *IDH1* | 4 | *SETBP1* | 4 |
| *CALR* | 9 | *IDH2* | 4 | *SF3B1* | 10-16 |
| *CBL* | 8,9 | *JAK2* | all | *SRSF2* | 1 |
| *CEBPA* | all | *KIT* | 2,8-11,  13,17 and18 | *TET2* | all |
| *CSF3R* | all | *KRAS* | 2,3 | *TP53* | all |
| *DNMT3A* | all | *MPL* | 10 | *U2AF1* | 2,6 |
| *ETV6* | all | *NPM1* | 10,11 | *WT1* | 6-10 |
| *EZH2* | all | *NRAS* | 2,3 | *ZRSF2* | all |

**Supplementary Table 3.** List of genes included in the Sophia genetics myeloid panel.

| N (%) | P/LP Mutations | N (%) | EuroMDS groups |
| --- | --- | --- | --- |
| 11 (14) | TET2 | 28 (48) | 0 |
| 10 (12) | SF3B1 | 3 (5) | 1 |
| 9 (11) | ASXL1 | 1 (2) | 2 |
| 7 (9) | SRSF2, DNMT3A | 3 (5) | 3 |
| 6 (7) | CEBPA | 5 (9) | 4 |
| 5 (6) | U2AF1 | 4 (7) | 5 |
| 4 (5) | ZRSR2, TP53 | 9 (15) | 6 |
| 3 (4) | CBL, IDH2, RUNX1, SETPB1 | 5 (9) | 7 |
| 2 (2) | KIT | | |
| 1 (1) | KRAS, EZH2, IDH1, ETV6 | | |

**Supplementary table 4**. Distribution of mutations according to EuroMDS groups. *P/LP: pathogenic/likely pathogenic.*

**Supplementary Figures**


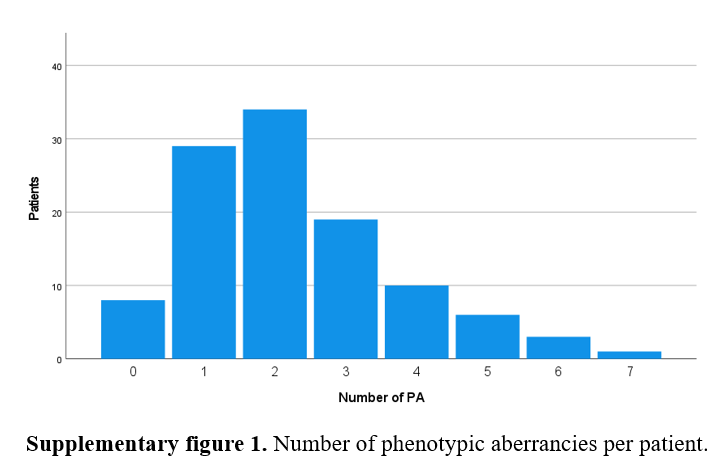


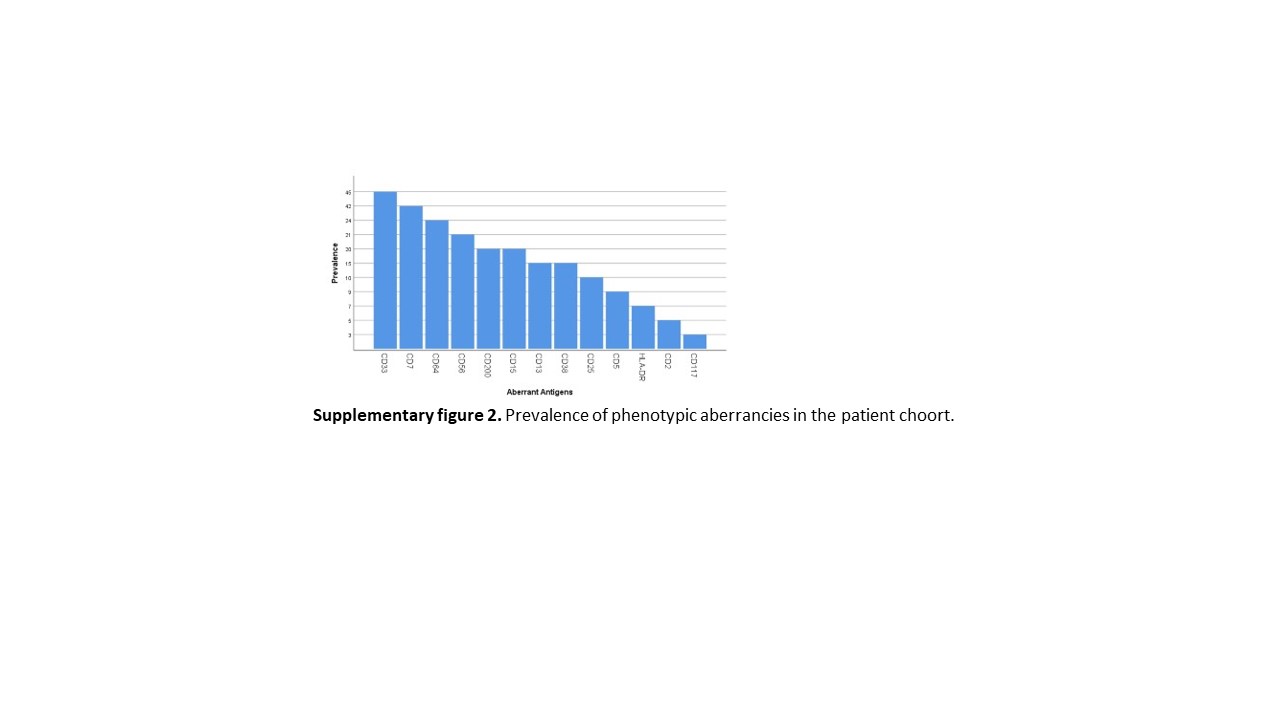


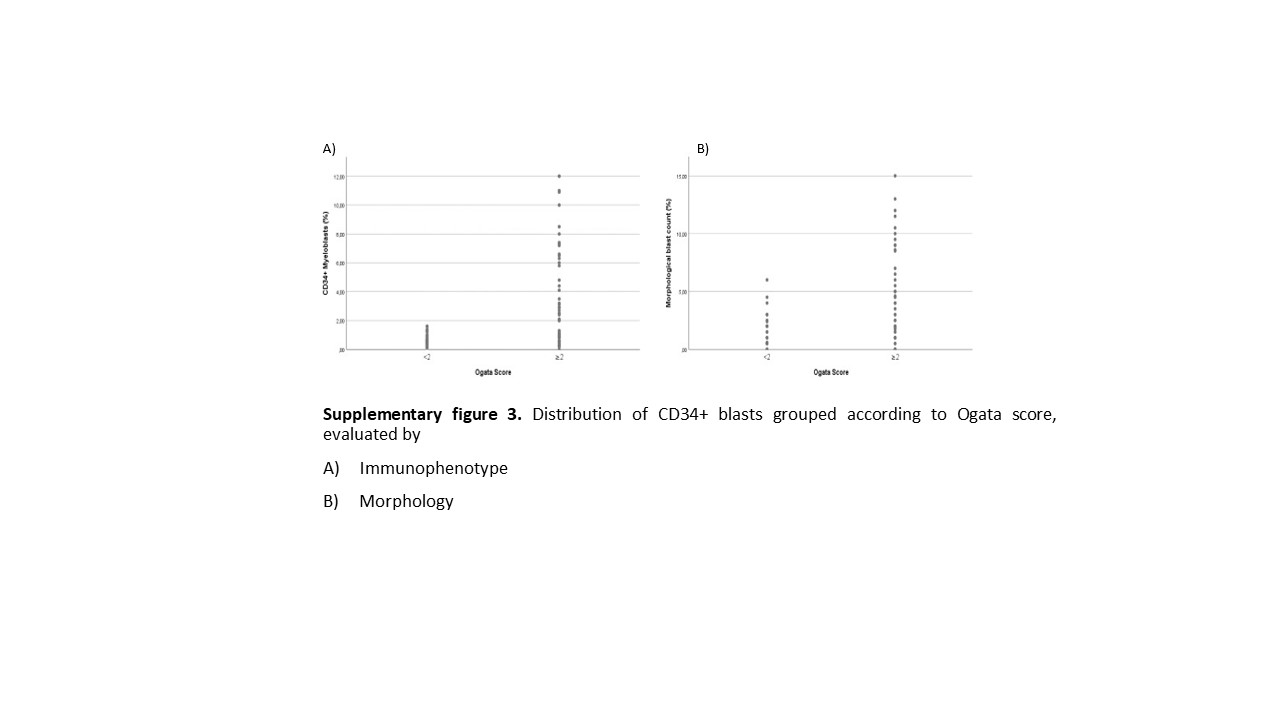


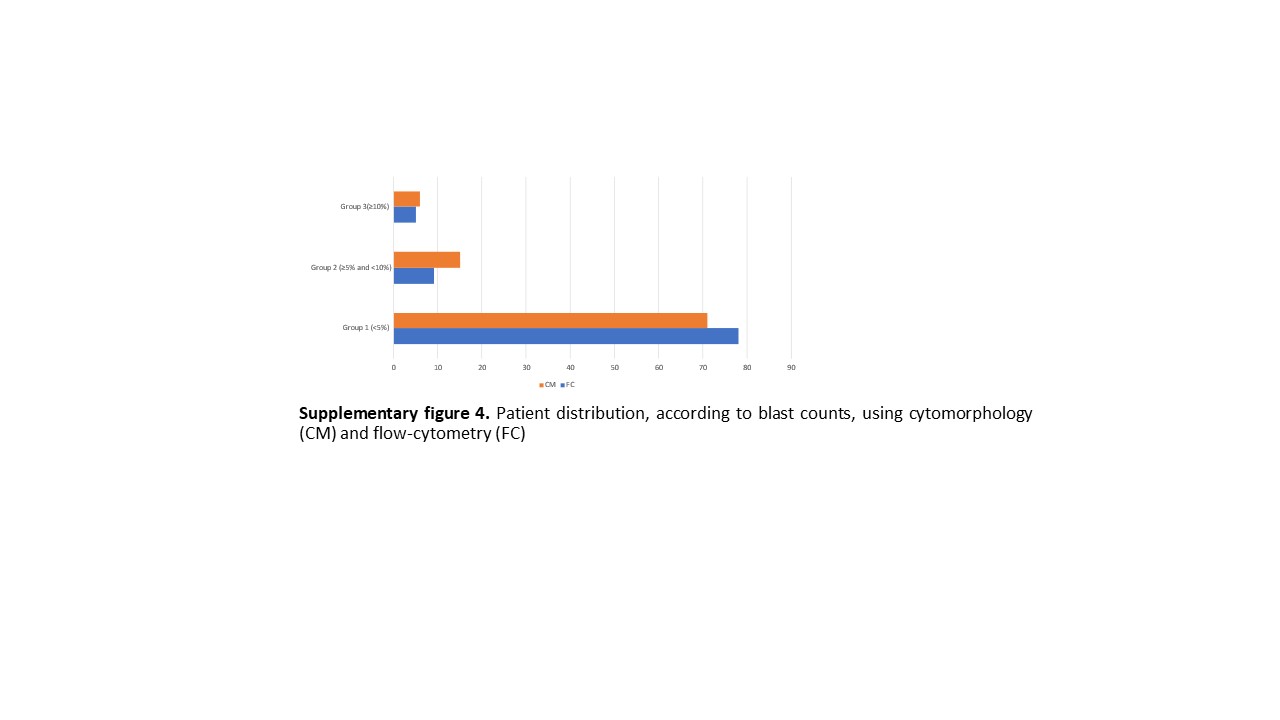


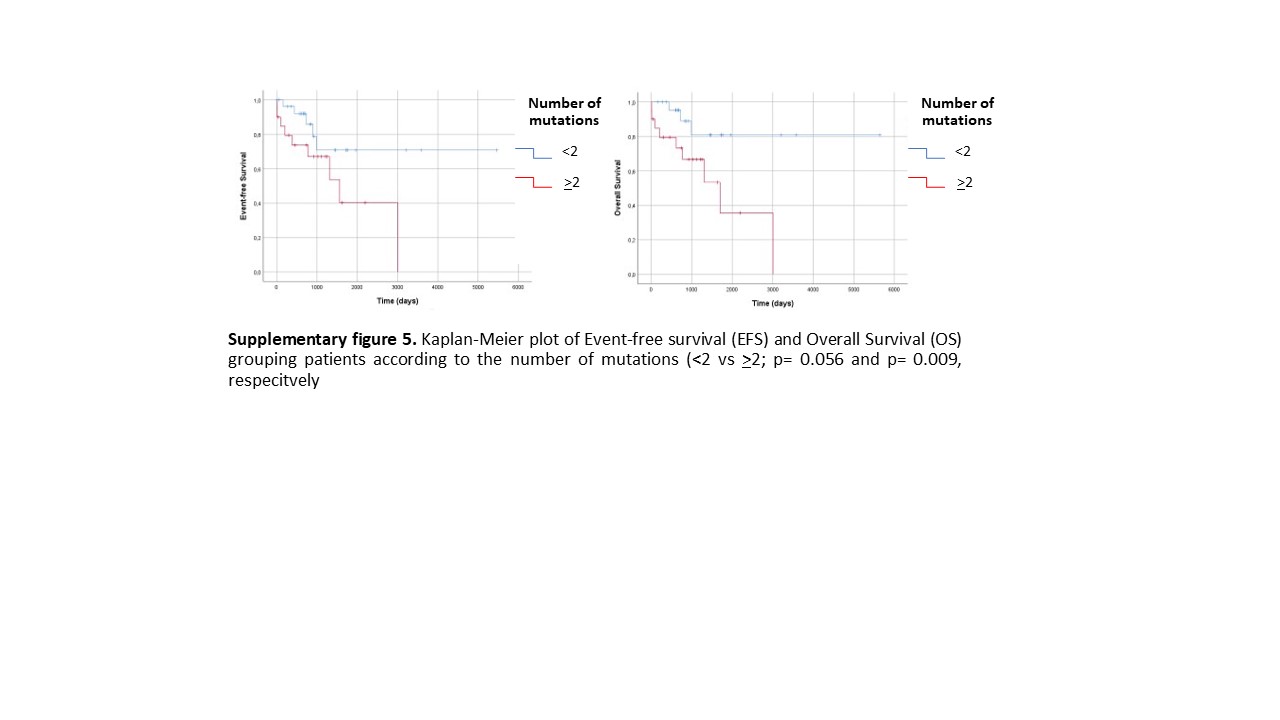


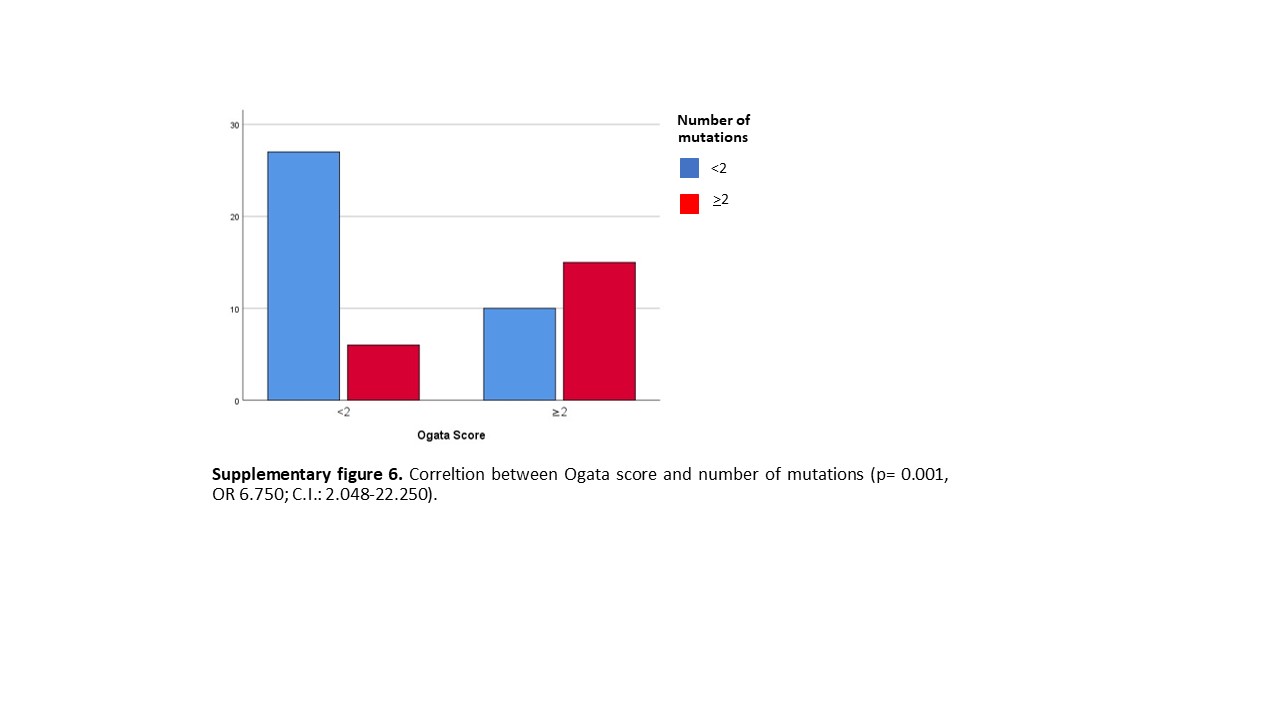

Supplement: Supplementary file 1 — (DOCX 250 kb) [file 277_2023_5384_MOESM1_ESM.docx]
